# Effects of educational intervention on knowledge of Full Outline of Un-Responsiveness score among health workers in uganda: a quasi -experimental pilot study

**DOI:** 10.1186/s12909-025-07839-8

**Published:** 2025-10-02

**Authors:** Obongo Tom, Kinkuhaire Betty, Tibaijuka Leevan, Kaddumukasa Mark, Nantongo Hanifah

**Affiliations:** 1 Department of Nursing, Mbarara University of Science and Technology, P.O. BOX 1410, Mbarara, Uganda; 2 Department of Obstetrics and Gynaecology, Mbarara University of Science and Technology, P.O. BOX 1410, Mbarara, Uganda; 3 Makerere University College of Health Sciences, P.O. BOX 7072, Kampala, Kampala Uganda; 4 Pallisa General Hospital, P.O. Box, 14, Pallisa, Uganda; 5 Department of Obstetrics and Gynaecology, Mbarara Regional Referral Hospital, P.O. Box 40, Mbarara, Uganda

**Keywords:** Full Outline Un-Responsiveness, Knowledge, Perception, Acceptability, Health workers

## Abstract

**Background:**

The Full Outline of Un-Responsiveness (FOUR) score is a new and better coma grading scale in critically ill patients. However, there is a paucity of data on its knowledge among health workers in sub-Saharan Africa. This study assessed the effects of an educational intervention on knowledge of FOUR Score among healthworkers at a Regional Referral Hospital in Southwestern Uganda.

**Methods:**

This was a one-group quasi-experimental study among conveniently sampled health workers attending to critically ill patients. Using a pretested questionnaire, data was collected pre and post intervention. Participants with low and moderate pre-test knowledge attended a theory session of 45-minutes and hands on sessions using the FOUR Score reference chart for one week, on ward. The post-test data was collected for all trained participants seven days after training. Data was analysed using STATA-17.

**Results:**

Of the 146 health workers (HWs) recruited, 50.7% and 45.2% were nurses and doctors, respectively. Over 113 (77.0%) were degree holders and 114 (78.1%) had no prior FOUR Score training. The median knowledge score was 8 (34.8%) and 19 (82.6%) in the pre- and post-test, respectively. A Wilcoxon Signed-Rank test showed a significant knowledge score difference in the median pre- and post-test (Z = 10.4; *p* < 0.001). Higher level of education (X2 [1] = 10.3, *P* = 0.016), working in ICU (X2 [2] = 13.8, *P* = 0.001), and prior FOUR Score training (Z = 6.3, *P* < 0.001) were significantly associated with high pre-test knowledge score but were not significant in the post-test.

**Conclusion:**

This educational intervention was effective in increasing the knowledge of healthworkers regarding the FOUR score, indicating that it can be easily learned and mastered. When properly done, this intervention could potentially improve the management of critically ill patient in similar (low- and middle-income countries) settings.

**Trial registration:**

Clinical traial number: not applicable.

**Supplementary Information:**

The online version contains supplementary material available at 10.1186/s12909-025-07839-8.

## Background

The Full Outline of Un-Responsiveness (FOUR) score, a validated clinical grading scale for evaluating altered level of consciousness, has attracted attention globally due to its ability to accurately assess the neurological status of intubated and aphasic patients [1, 2] and might challenge the Glasgow Coma Scale (GCS) as a gold standard in the near future [3, 4].

The GCS is generally a popular scoring system for altered levels of consciousness because it is theoretically simple, easy to use, included in available guidelines and preprinted on patient charts [5]. However, even with all the modifications, GCS still has limitations and inaccuracies among certain patient populations [6, 7]. The GCS drawbacks include, among others, the inability to evaluate verbal response in intubated patients, items not equally weighted, lacks respiratory and brainstem reflexes component [7, 8].

To address the pitfalls of the GCS, the FOUR score was devised and first validated in 2005 by Wijdicks and his colleagues at Mayo Clinic as an accurate option [9]. The FOUR Score is a seventeen-point scale, comprising of four equally weighted components or categories: eye response, motor response, brainstem reflexes, and pattern of respiration. Each component of FOUR Score is a 5-point scale, ranging from 0 to 4, with the summed score ranging from 0 to 16 [9, 10], with the score of 16 being the highest level of consciousness [11]. A diagnosis of imminent brain death (IBD) is reached when the total FOUR Score is 0 [12].

Impaired Level of Consciousness remains a common acute medical problem frequently seen in casualties following traumas as well as at acute care units [13]. The European Academy of Neurology Guideline on diagnosis of coma and other disorder of Unconsciousness recommends that FOUR score be used in acute setting instead of GCS [14].

The FOUR score can be reliably applied by health workers with different levels of education and varying experiences after training on it, ultimately leading to enhanced communication about critical information, rapid team response and improved overall patient outcomes [5, 15, 16]. A simple structured training on FOUR Score beforehand increases health workers’ knowledge and encourages the use of this coma scale [17–19].

Recent evidence on validation of FOUR score among different patient populations and its inter-rater reliability plus predictive ability compared to GCS in several countries, including Uganda, is excellent [1, 3–5, 8, 9, 13, 16, 20–24].

With all the known effectiveness and benefits of the FOUR score, few healthworkers and units caring for critically ill patients at Mbarara Regional Referral Hospital (MRRH) use it, this ultimately affects optimal patient outcomes. In addition, there is a paucity of data on knowledge of HealthCare workers (HCWs) regarding the FOUR score in the study setting. Mbarara Regional Referral Hospital has a high influx of patients with neurological lesions like stroke and traumatic brain injury as it serves a very big catchment area in Southwestern Uganda. In addition, it is a teaching hospital for Mbarara University of Science and Technology (MUST), and other health institutions. The hospital offers tertiary preventive, diagnostic, curative, emergency, and rehabilitative services. It also serves as an internship site for medical interns who have graduated from different universities in Uganda. Hence, this study aimed at assessing effects of educational intervention on knowledge among health workers at MRRH.

## Methods

### Study design

This study employed a one-group prospective Quasi-experimental design, using a quantitative method of data collection.

### Study population

Healthworkers caring for adult acute and critically ill patients in the Intensive Care Unit (ICU), Accident and Emergency Unit (A&E), and surgical and medical wards at MRRH were recruited (qualified nurses of all cadres, interns medical officers, senior house officers (SHOs), physicians, surgeons physiotherapists, and medical clinical officers).

### Inclusion criteria

All healthworkers [medical clinical officers, interns (nurses and doctors), qualified nurses of all cadres, medical officers, physicians, surgeons, and SHOs (surgery, anesthesiology, internal medicine)] working at the ICU, accident and emergency unit, and surgical and medical wards during the time of data collection. Participants were included in the training phase if they scored 75% or below in pre-test. Participants proceeded to the post-test knowledge phase if they attended the pretest and training session.

### Exclusion criteria

Potential participants who were on any form of leave during the data collection period.

### Sampling method

All qualified health workers who worked on the selected units and met the inclusion criteria during the data collection period were recruited.

### Sample size

The estimated target population (N) was 155 (Human Resource Department MRRH, January 2023). Using the Yamane formula, Sample size (n) = N/1 + N(e^2^). Where; N = Target population, e = type 1 error. *n* = 155/1 + 155(0.05^2^) = 112. Plus 10% attrition rate = 11, *n* = 112 + 11 = 123. However, we recruited 146 participants for pretest and in post-test, sample size was less by those who had high baseline score. A post hoc analysis at a 95% Confidence level, a type 1 error of 0.05, a sample size of *n* = 146, and an effect size of 0.5, the achieved actual power of the studty was 0.99.

### Study variables

#### Dependent variables

The main explanatory variable for this study was knowledge (definition, indication, components, scoring range, limitation, rationale for using the score, and how to assess the score) of the FOUR Score among health workers [19].

#### Independent variables

The demographic characteristics of the health workers, including profession, current unit, and level of education (categorized into certificate, diploma, degree, & postgraduate); clinical experience in years; and prior training on four scores categorized into “yes” and “no”.

#### Data collection tools

A self-administered questionnaire written in English was used to gather data on knowledge of the FOUR Score among health workers. The questionnaire comprised two parts: Part *A - demographic* characteristics of participants to facilitate meaningful analysis and interpretation the results; Part *B - knowledge* of the FOUR Score among health workers. The questions consisted of definition, indication, components, scoring range, limitations, and how to score on a FOUR Score. The questionnaire (suplementary material 1) [25], was developed and validated for this study after reviewing the related literature [19] and the questions were similar in both pre- and post-educational intervention.

#### Data collection procedure

The researcher sought clearance to collect data from the hospital director and then used convenient methods to enroll health workers in the study. Data was collected in two phases; the first phase was pre-intervention knowledge of FOUR Score which lasted two weeks, followed by one week training of health workers on FOUR Score, then phase two data collection, started two weeks apart from phase one and took two weeks.

During the pre-intervention phase (3^RD^ & 4^TH^ weeks of January 2023), participants filled out the questionnaire that assessed baseline knowledge of healthworkers regarding the FOUR Score.

An educational intervention Participants with a low to moderate level of knowledge (0 to 74%) of the FOUR Score in the pretest attended a 45-minute formal educational session in the 1^ST^ week of February 2023, consisting of Microsoft PowerPoint (15 slides) and the FOUR Score chart, conducted either in small groups or individually in the participating units/wards. The training was scheduled outside work schedules to avoid duty disruptions. Over the course of one week, the researcher and research assistants encouraged health workers on duty in the participating units to assess the neurological responsiveness of patients using the FOUR Score. Participants’ contact details were obtained to remind them of their scheduled post-test. Each participating unit was provided with a FOUR Score chart for reference. Participants with higher baseline knowledge were excluded from the training phase, posttest and analysis.

**Fig. 1:**
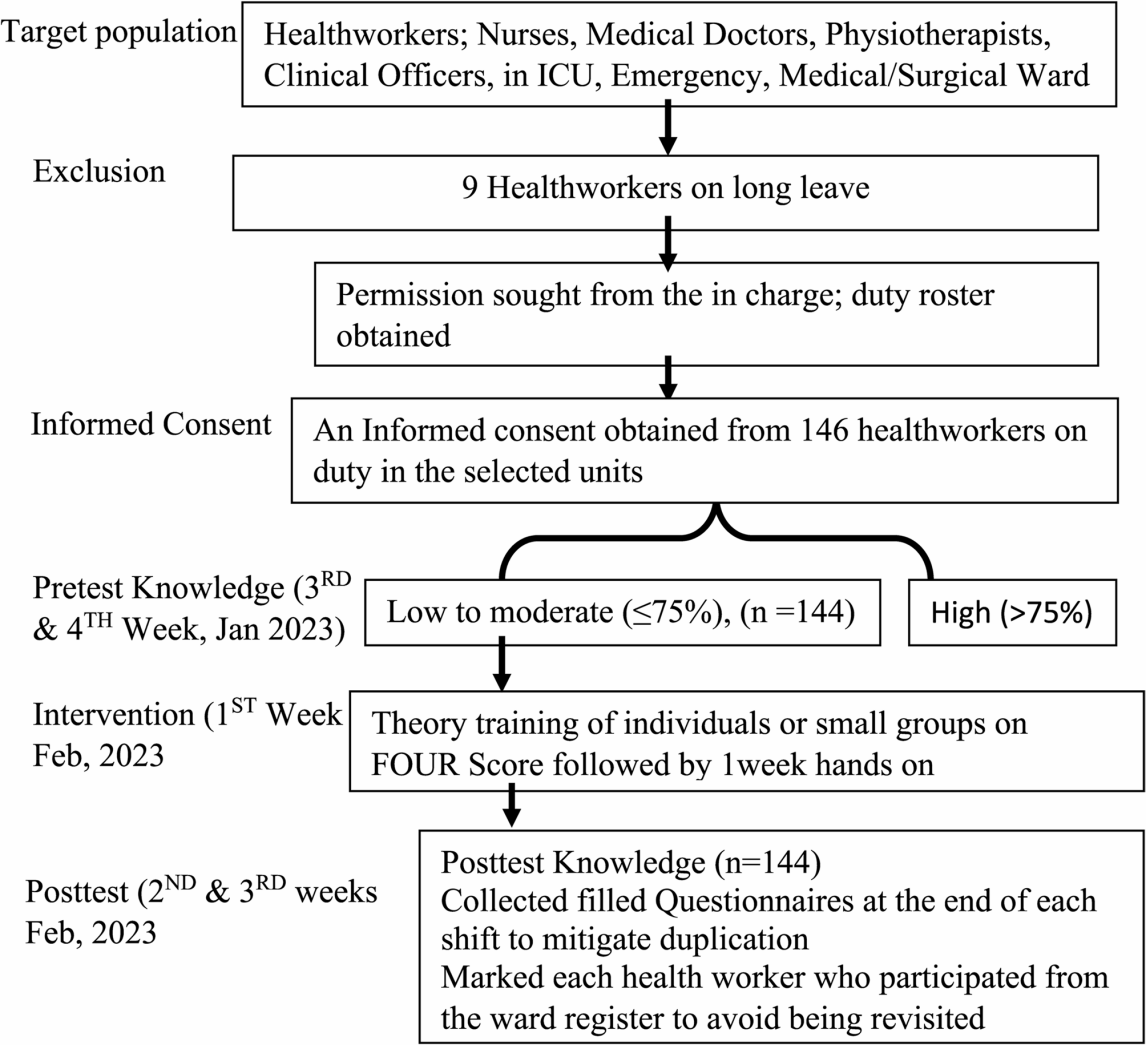
The schematic for knowledge of FOUR score data collection (5 weeks)

Post-intervention data collection The researcher and research assistants identified trained health workers, who then completed a paper-based post-intervention self-administered questionnaire in English on knowledge of the FOUR Score. Post-tests were done one week after the training on FOUR Score to ensure a uniform period of exposure. Post-test data collection was done over a period of two weeks (2^ND^ & 3^RD^ weeks of February 2023). See Fig. 1 below;

### Data analysis

Data was entered in Redcap and exported to STATA version 17 for data cleaning and analysis. Descriptive statistics like, mean, median, and standard deviations for numerical data, and then frequencies and percentages for categorical data were obtained. The total percentage test score of 0–49% was taken as a low level of knowledge, 50–75% was considered moderate, and a score above 75% was considered a high level of knowledge about FOUR score. This cutoff is similar to that used in Palestine [26]. An overall median knowledge level about FOUR Score was calculated for both pre- and post-educational intervention out of the maximum score points. A normality tests was performed. A Wilcoxon Signed-Rank Test using 144 participants, Mann-Whitney U tests and Kruskal-Wallis tests were used to explore the pre-tests and post-tests scores.

### Quality control

*Validity* was achieved by pre-testing the questionnaires at Mayanja Memorial Hospital and also involved two experts in Critical Care Nursing and Research who reviewed and validated the structured questionnaires for suitability.

The calculated Scale-Content Validity Index Average (S-CVI Ave) of the questionnaires, based on Universal Agreement (UA) Average and Item Content Validity Index (I-CVI), was **0.94 and 0.97**, respectively. The questionnaire achieved a statisfactory content validity (at least 0.8) when two experts are used [27].

Two research assistants who had certificates in responsible conduct of research (RCR) were trained before participating in the data collection exercise.

Reliability Experts in critical care confirmed the consistency of the questionnaire (*internal reliability and expert judgment*) before taking it to the field for piloting.

### Ethical consideration

Ethical clearance was obtained from Research Ethic Committee (REC) of MUST (MUST-2022-645). Administrative clearance was obtained from MRRH director. The Helsinki Declaration on research involving human subjects was adhered to throughout the research process.

## Results

Table 1 shows that out of 146 recruited HCWs, the majority (71, 48.6%) were aged 30–40 years, and the median age was 32 (range of 24–55, IQR = 10 years). The majority (74, or 50.68%) and 66, or 45.21%) were nurses and doctors, respectively. By qualification, the median years of clinical experience was 5 years, over 113 (77%) had at least a degree. Most HCWs (114, 78.1%) had not received any prior training on FOUR Score.

**Table 1:** Health worker’s demographic characteristics

Characteristic		Frequency (%), *n* = 146
Age category (years)	20 to < 30	5 (35.6)
	30 to < 40	71 (48.6)
	Above 40	23(15.7)
Gender	Male	84 (57.5)
	Female	62 (42.5)
Cadre	Nursing	74(50.68)
	Doctors	66 (45.21).
	Physiotherapy	4 (2.74)
	Medical clinical officers (MCO)	2 (2.05)
Level of education	Certificate (Nurses)	9 (6.2)
	Diploma (Nurses + MCO)	24 (16.4)
	Bachelor’s/Degree	54 (37)
	Postgraduate/Residents	59 (40.4)
*Postgraduate specialty*:	Critical care nursing	21 (36)
	Emergency medicine	6 (10)
	Surgery	9 (16)
	Internal medicine	10 (17)
	Anaesthesia and critical care	11 (19)
	Physiotherapy	2 (3)
Clinical experience	Less than 5 years	66 (45.2)
	5 to 10 years	47 (32.2)
	More than 10 years	33 (22.6)
Currently unit	Intensive Care Unit	34 (23.3)
	Accident and Emergency Unit	54 (37.0)
	Medical-Surgical general ward	58 (39.7)
Duration of work in the current unit:	Below 3 months	46 (31.5)
	4 to 11 months	30 (20.5)
	1 to 2 years	30 (20.5)
	Above 2 years	40 (27.4)
Prior training on FOUR score:	No	114 (78.1)
	Yes	32 (21.9)
Place of prior training on FOUR	From medical/nursing school	16 (50)
	In-service training	16 (50)

### Knowledge of FOUR score among healthcare workers

Normality tests (Shapiro-Wilk test) had a significant *p*-value of 0.01 and < 0.001 for pretest and post-test total percent knowledge scores, respectively.

Table 2 above presents the total percent knowledge of health workers on FOUR Score before and after an educational intervention. Participants had low knowledge of FOUR Score before training, with a median score of 8 out of 23 items (34.8%), ranging from 13.04 to 82.6% and an Interquartile Range (IQR) of 34.8%. After an educational intervention, there was high knowledge on FOUR Score, with median score of 19 (82.6%), ranging from 39.1 to 87.0%, and an IQR of 2 (8.7%). A Wilcoxon Signed Rank Test (*N* = 144 participants) showed a statistically significant difference in median scores between pretest and post-test (z = 10.4, *p* < 0.0001). There were 143 observations for which post-test was greater than pretest total percent knowledge score.

**Table 2:** The distribution of healthcare workers regarding their knowledge of FOUR score

		PRETEST (*n* = 146)	POST TEST(*n* = 144)
Knowledge level of FOUR Score	Median score (%)	8 (34.78%)	19 (82.61%)
Knowledge categories	Low knowledge (< 50%)	104(71.2%)	2(1.4%)
	Moderate knowledge (50% to75%)	40(27.4%)	31(21.5%)
	High knowledge (> 75%)	2(1.4%)	111(77.1%)
Knowledge Domain	Definition of FOUR score	63(43.2%)	132(91.7%)
	Component of FOUR score	38(26.0%)	130(89.0%)
	Brainstem reflexes	30(20.6%)	126(86.3%)
	Indication	85(58.2%)	137(95.1%)
	Range	55 (37.7%)	131(91.0%)
	Rationale	4 (2.7%)	88(61.1%)
	How to score (high knowledge, 7 to 9 scores).	12 (8.2%)	121(84.0%)

Table 3 above presents a relationship between healthworkers knowledge of FOUR Score and their demographic characteristics. In the pre-test, though HCWs generally had low knowledge of FOUR Score, those with a higher level of education had statistically higher knowledge of FOUR X2(3) = 10.33, *P* = 0.016). In relation to the unit of current work or rotation, those working in ICU had significantly higher baseline knowledge of FOUR Score X2(2) = 13.81, *P* = 0.001). FOUR Score had higher baseline knowledge about it compared to those who had not (***z*** = 6.308; *P* < 0.001), irrespective of whether they were trained in medical or nursing schools or in-service.

**Table 3:** Knowledge of FOUR score versus characteristics of participants

		Pre-test median		Post-test median	
Age category	20 -<30	37.0%	X2(3) = 0.85, *P* = 0.655	82.6%	X2(3) = 3.78, *P* = 0.15
	30-<40	39.13%		82.61%	
	> 40	30.43%		82.61%	
Education level	Certificate	17.39%	X2(3) = 10.33, *P* = 0.016	73.9%	X2(3) = 5.98, *P* = 0.113
	Diploma	30.43%		79.3%	
	Degree	30.43%		80.9%	
	Post-grad	43.48%		82.6%	
Clinical experience (years)	< 5	30.43%	X^2^_(2)_ = 1.07, *P* = 0.59	82.61%	X2(2) = 0.13, *P* = 0.940
	5–10	43.48%		82.61%	
	> 10	30.43%		82.61%	
Current ward	ICU	54.35%	X^2^_(2)_ = 13.81, *P* = 0.001	82.61%	X^2^_(2)_ = 1.54, *P* = 0.470
	A&E	30.43%		82.61%	
	Medicine/Surgery	30.43%		82.61%	
Prior training on FOUR Score	No	30.43%	**z** = 6.308; *P* < 0.001	82.61%	z = 0.65; *P* = 0.520
	Yes	58.7%		82.61%	

## Discussion

The present study set to assess the knowledge of FOUR score among healthcare workers before and after an education intervention at a Tertiary Referral Hospital in Southwestern Uganda. Healthworkers caring for critically ill patients must be knowledgeable about the different neurological assessment tools [28]. Generally, Healthcare workers had low baseline knowledge of FOUR Score and the educational intervention had a positive effect on healthworkers’ knowledge and bridged the critical training gaps in Neurological assessment. This finding proves that a focused, structured educational interventions, however short, can significantly improve the knowledge of healthcare workers regarding FOUR score especially in resource limited settings like Ugandan tertiary Hospitals. Similar findings of low baseline knowledge have been reported among ICU Nurses by other researchers [18, 19, 29], attributed to lack of familiarity with the scale, as it is relatively new and not included in most guidelines and preprinted patient’s charts. The low pre-test knowledge among the participants underscores the need for formal training on all neurological assessment scales and demonstrates the potential for impactful learning strategies in resource constrained settings. On the other hand, the result confirms the critical clinical training gap and shortage of neurocritical trained HCWs in low resource settings, they are general nurses/practitioners, which calls for a wider systematic approach [23, 28]. The low pre-test knowledge suggests that FOUR score is not routinely taught in Ugandan Nursing and medical curricula. This calls for policy and curriculum reforms by incorporating this coma scale into the undergraduate programs (physiotherapy, nursing, medical clinical officers), advanced critical care education (like; Intensive Care nursing, anesthesia, internal medicine, Surgery), and in-service refresher trainings. Report from Egypt indicate similar gaps resulting from lack of formal instructions [19].

Many of the nurse participants had lower qualifications (Diploma and certificate) that lack emphasis on critical care nursing and often have truncated professional training coupled with lack of resources and educational support to effectively manage critically ill patients.

The post graduate students are undertaking specialized training in managing critically ill patients, this exposes them to different grading scales, thus the high scores. This is contrary to [18] report, that level of education had no correlation with the knowledge of FOUR score among the 49 nurse participants. Health workers with specialty training, are more knowledgeable about neurological assessment scales than staff nurses in ICU with a more generalized qualification [16]. This underscores the significance of specialty training by healthworkers caring for critically ill patients.

Our study also noted that, health workers in the ICU had significantly higher knowledge of FOUR Score than those in other units. This was only statistically significant before training. This contradicts Baraka [19], who reports that healthworkers at emergency units have significantly higher knowledge on FOUR Score than ICU staff. This difference in baseline knowledge about the FOUR score by ward is due to the difference in familiarity, as ICU and A&E staff use it more frequently than health workers in the general wards [30].

Healthworkers with prior training on FOUR Score had significantly higher knowledge about it compared to those who had not, irrespective of whether they were trained in medical or nursing schools or in-service. Previous training on the FOUR Score has a higher positive association with high knowledge of nurses on it [19, 21]. The result underscores the importance of continuous professional development (CPD) for health workers and the need to include FOUR scores in the syllabus for novice healthworkers. It further provides proof that the FOUR score can easily be learnt by all cadres with different qualifications.

The current study finding also agree with reports that health workers with varying levels of clinical experience can learn and reliably evaluate patients using FOUR Score if they are trained on it [5, 15, 18].

The 45-minute theory plus one-week practical training model was effective in the short term. In Egypt [18], noted that two training sessions, each lasting 40 to 50 min, using lecture, group discussion, demonstration and return remonstration as teaching method, with the medium of instruction being Handouts and audiovisual, significantly improve nurses knowledge, practice and self-confidence on GCS and FOUR score. Likewise, using a combination of teaching methods and medium above, over 4-hours, effectively improved the knowledge of both FOUR score and GCS among Jordan’s ICU Nurses [28].

However, studies show that trainings held repeatedly or over a longer period ensures sustained competency. Simulations-based learning improve knowledge, critical care skills including neurological assessment, and retention after five months [31–33], but optimization of learning and long-term retention, can be achieved through a blended learning approaches that involves multiple touchpoints.

A combined format involving e-learning modules and hands-on sessions, can address scheduling constraints of in-service refresher trainings while enhancing the learning outcome for healthcare workers in busy settings.

Scaling up this training model, to make FOUR Score training work Nationally, requires a multi-level collaboration. The Ministry of Health (MOH) could integrate this Coma scale into the National emergency and critical care guidelines, and preprinted charts just like the GCS. Training Nurses, clinical officers to acts as FOUR score champions, could decentralize expertise and improve knowledge of the scale across the health care system.

### Study strengths and limitation

All health workers working/rotating in ICU, A&E, medical and Surgical wards, involed in the clinical management of adult critically ill patients were included in the study. The data collection tools used, achieved a satisfactory level of content validity.

While the study shows promising outcomes, we acknowledge its limitations. It was conducted at a single center (a regional referral teaching hospital in western Uganda), assumed to represent government aided health facilities in terms of the care provided. However, others health facilities could be operating under different conditions, hence, similar intervention should be tested in district hospitals and lower Health centers, where most critically ill patients are first managed. However, since majority of the participants were postgraduate residents and interns who work or will work in those different hospitals respectively, the findings may be generalizable.

The study employed convenient sampling technique to recruit participants, which is limited in terms of representativeness. This was addressed by recruiting all the health workers who worked or rotated at the participating wards during the data collection period.

The 45 min of theory session followed by one week of Hands-on, was brief, to determine the long-term impact of the intervention. Future studies should carryout assessment at 3 and 6 months to evaluate knowledge retention [18].

## Conclusions

This study confirmed that focused, short-duration educational intervention can effectively increase the knowledge of health workers regarding the FOUR score, indicating that it can be easily learned and mastered. However, long-term retention, and improvement in clinical outcomes require policy integration, refressher training sessions, and accessible, scalable delivery formats. Training clinical officers and Nurses at district hospital and rural Healthcenters can improve its scalability. By embedding the FOUR score into national training frameworks, clinical guidlines, promoting blended learning, Uganda and similar LMICs can strengthen their neurological assessment capacity in critical care settings. Future research should focus on retention sttrategies and cost effective training models to ensure sustained impact.

## Supplementary Information

Supplementary Material 1.

Supplementary Material 2.

## Data Availability

The datasets used and/or analyzed during the current study are available under the supplementary files or from the corresponding author on reasonable request.

## Abbreviations

A&E: Accident and Emergency unit
CINAHL: Cumulative index for Nursing and Allied Health Literature
FOUR: Full Outline of Un-Responsiveness
GCS: Glasgow Coma Scale
HDU: High Dependency Unit
HCWs: HealthCare Workers
ICU: Intensive Care Unit
IQR: Interquartile Range
KPA: Knowledge, Perception and Acceptability
LMIC: Low and Middle Income Countries
LOC: Level of Consciousness
MRRH: Mbarara Regional Referral Hospital
MUST: Mbarara University of Science and Technology
TBI: Traumatic Brain Injury

## References

[1] Jamal A, Sankhyan N, Jayashree M, Singhi S, Singhi P. Full Outline of Unresponsiveness score and the Glasgow Coma Scale in prediction of pediatric coma. World J Emerg Med. 2017;8(1):55. https://pmc.ncbi.nlm.nih.gov/articles/PMC5263038/10.5847/wjem.j.1920-8642.2017.01.010 PMC5263038 28123622

[2] Wijdicks EF, Bamlet WR, Maramattom BV, Manno EM, McClelland RL. Validation of a new coma scale: the FOUR score. Annals of Neurology: Official Journal of the American Neurological Association and the Child Neurology Society. 2005 Oct;58(4):585-93. 10.1002/ana.20611 10.1002/ana.20611 16178024

[3] Gujjar AR, Jacob PC, Nandhagopal R, Ganguly SS, Obaidy A, Al-Asmi AR. Full Outline of UnResponsiveness score and Glasgow Coma Scale in medical patients with altered sensorium: interrater reliability and relation to outcome. J Crit Care. 2013 Jun 1;28(3):316-e1.10.1016/j.jcrc.2012.06.009 10.1016/j.jcrc.2012.06.009 22884530

[4] Hickisch A, Holmefur M. Swedish translation and reliability of the Full Outline of Unresponsiveness Score. J Neurosci Nurs. 2016 Jul 1;48(4):195-205. https://journals.lww.com/jnnonline/abstract/2016/08000/swedish_translation_and_reliability_of_the_full.6.aspx 10.1097/JNN.0000000000000205 27224684

[5] Almojuela A, Hasen M, Zeiler FA. The Full Outline of UnResponsiveness (FOUR) Score and its use in outcome prediction: a scoping systematic review of the adult literature. Neurocrit Care. 2019 Aug 15;31(1):162-75. 10.1007/s12028-018-0630-9 10.1007/s12028-018-0630-9 30411302

[6] Sepahvand E, Jalalı R, Mırzaeı M, Ebrahımzadeh F, Ahmadı M, Amraıı E. Glasgow Coma Scale versus full outline of UnResponsiveness Scale for prediction of outcomes in patients with traumatic brain injury in the intensive care unit. Turk Neurosurg. 2016. https://www.eprints.lums.ac.ir/563/. 10.5137/1019-5149.JTN.13536-14.0 27476914

[7] Bajaj J, Yadav Y, Sharma D. Modifications of Glasgow coma Scale—a systematic review. Indian J Surg. 2023;85(5):1023–34.

[8] Jalali R, Rezaei M. A comparison of the Glasgow Coma Scale score with full outline of unresponsiveness scale to predict patients’ traumatic brain injury outcomes in intensive care units. Crit Care Res Pract. 2014;2014(1):289803. 10.1155/2014/289803 10.1155/2014/289803 PMC4071859 25013727

[9] Wijdicks EF, Bamlet WR, Maramattom BV, Manno EM, McClelland RL. Validation of a new coma scale: the FOUR score. Annals Neurology: Official J Am Neurol Association Child Neurol Soc. 2005;58(4):585–93. 10.1002/ana.20611 16178024

[10] McNett M, Amato S, Gianakis A, Grimm D, Philippbar SA, Belle J, Moran C. The FOUR score and GCS as predictors of outcome after traumatic brain injury. Neurocrit Care. 2014 Aug;21(1):52-7. 10.1007/s12028-013-9947-6 10.1007/s12028-013-9947-6 24408147

[11] Foo CC, Loan JJ, Brennan PM. The relationship of the FOUR score to patient outcome: a systematic review. J Neurotrauma. 2019;36(17):2469–83. 31044668 10.1089/neu.2018.6243 PMC6709730

[12] Zappa S, Fagoni N, Bertoni M, Selleri C, Venturini MA, Finazzi P, Metelli M, Rasulo F, Piva S, Latronico N, Imminent Brain Death (IBD) Network Investigators. Determination of Imminent brain death using the full outline of unresponsiveness score and the glasgow coma scale: a prospective, multicenter, pilot feasibility study. J Intensive Care Med. 2020 Feb;35(2):203-7.10.1177/0885066617738714 10.1177/0885066617738714 29084482

[13] Shalaby S, Reda NA, Emam NO. Full Outline of Un-Responsiveness Scale (FOUR) versus modified Glasgow Coma Scale (GCS) in predicting discharge outcomes of altered consciousness patients, (January). Am J Nurs Res. 2019 Jan 3;7(1):79-86. https://pubs.sciepub.com/ajnr/7/1/11/

[14] Kondziella D, Bender A, Diserens K, van Erp W, Estraneo A, Formisano R, et al. European academy of neurology guideline on the diagnosis of coma and other disorders of consciousness. Eur J Neurol. 2020;27(5):741–56. 32090418 10.1111/ene.14151

[15] Anestis DM, Tsitsopoulos PP, Foroglou NG, Tsatali MS, Marinos K, Theologou M, Tsonidis CA. Cross-cultural adaptation and validation of the greek version of the “Full Outline of Unresponsiveness Score”: a prospective observational clinimetric study in neurosurgical patients. Neurocrit Care. 2022 Apr;36(2):584-94. 10.1007/s12028-021-01342-w 10.1007/s12028-021-01342-w PMC8460202 34558023

[16] Kondziella D, Bender A, Diserens K, van Erp W, Estraneo A, Formisano R, Laureys S, Naccache L, Ozturk S, Rohaut B, Sitt JD. European Academy of Neurology guideline on the diagnosis of coma and other disorders of consciousness. Eur J Neurol. 2020 May;27(5):741-56. 10.1111/ene.14151 10.1111/ene.14151 32090418

[17] Sepahvand E, Jalalı R, Mırzaeı M, Ebrahımzadeh F, Ahmadı M, Amraıı E. Glasgow Coma Scale versus full outline of UnResponsiveness Scale for prediction of outcomes in patients with traumatic brain injury in the intensive care unit. Turk Neurosurg. 2016. http://eprints.lums.ac.ir/563/10.5137/1019-5149.JTN.13536-14.0 27476914

[18] Loutfy A, Elzeiny A, Sabek EM, El-Monshed AH, Shahin MAH, Mohamed FSA. Effect of an educational program on pediatric nurses’ knowledge, practice, and self-confidence about level of consciousness scales. J Pediatr Nurs. 2023;73:e570–8. 37926670 10.1016/j.pedn.2023.10.035

[19] Abd Elrazek Baraka A, A Shalaby S. Effect of training sessions about Full Outline of Un-Responsiveness scale compared to Glasgow Coma Scale on nurses’ performance, perception and its reliability. Egypt J Health Care. 2021 Mar 1;12(1):54-72. https://journals.ekb.eg/article_135122_93f43d97f9b6881d95e280670f2850ef.pdf

[20] Stead LG, Wijdicks EF, Bhagra A, Kashyap R, Bellolio MF, Nash DL, et al. Validation of a new coma scale, the FOUR score, in the emergency department. Neurocrit Care. 2009;10:50–4. 18807215 10.1007/s12028-008-9145-0

[21] Czaikowski BL, Liang H, Stewart CT. A pediatric FOUR score coma scale: interrater reliability and predictive validity. J Neurosci Nurs. 2014 Apr 1;46(2):79-87. 10.1097/JNN.0000000000000041 10.1097/JNN.0000000000000041 24556655

[22] Fischer M, Rüegg S, Czaplinski A, Strohmeier M, Lehmann A, Tschan F, Hunziker PR, Marsch SC. Inter-rater reliability of the Full Outline of UnResponsiveness score and the Glasgow Coma Scale in critically ill patients: a prospective observational study. Crit Care. 2010 Apr 14;14(2):R64. 10.1186/cc8963 10.1186/cc8963 PMC2887186 20398274

[23] Abdallah A, Demaerschalk BM, Kimweri D, Aden AA, Zhang N, Butterfield R, et al. A comparison of the full outline of unresponsiveness (FOUR) and Glasgow coma scale (GCS) scores in predicting mortality among patients with reduced level of consciousness in Uganda. Neurocrit Care. 2020;32:734–41. 31392656 10.1007/s12028-019-00806-4 PMC7004860

[24] Aditya F. Differences of the Glasgow coma scale and the full outline of unresponsiveness scores on consciousness level examination. Jurnal Penelitian Perawat Profesional. 2020;2(4):545–54.

[25] Obongo T, Kinkuhaire B, Tibaijuka L, Kaddumukasa M, Hanifah N, Kinkuhaire B, et al. Questionnaire on knowledge of Healthcare workers on Full Outline of Un-Responsiveness score. 2024 *(unpublished).*

[26] Alnawafleh A, Alaraj H. The effectiveness of training sessions about Glasgow coma scale (GCS) and full outline of Un-Responsiveness (FOUR) score on Jordanian ICU nurses’ knowledge and perception. Palestinian medical and pharmaceutical journal. (Pal Med Pharm J). 2024;9999(9999):None–None.

[27] Davis LLJAnr. Instrument review: getting the most from a panel of experts. 1992;5(4):194–7.

[28] Prust ML, Mbonde A, Rubinos C, Shrestha GS, Komolafe M, Saylor D, et al. Providing neurocritical care in resource-limited settings: challenges and opportunities. Neurocrit Care. 2022;37(2):583–92. 35840824 10.1007/s12028-022-01568-2

[29] Bamani M. Study to Assess the Effectiveness of Structured Teaching programme on Knowledge regarding four score (full outline of unresponsiveness) coma scale among Staff Nurses working in Intensive care units in selected Hospitals. Int J Nurs Educ Res. 2021 Mar 31. https://ijneronline.com/HTML_Papers/International%20Journal%20of%20Nursing%20Education%20and%20Research__PID__2021-9-1-18.html

[30] Yglesias SII, NURSES’AWARENESS AND PERCEPTION OF FOUR SCORE SCALE, VERSUS GLASGOW COMA SCALE TOOL USED AMONG INTUBATED PATIENTS. Malaysian J Nurs (MJN). 2020;12(1):63–72.

[31] Han SG, Kim YD, Kong TY, Cho J. Virtual reality-based neurological examination teaching tool (VRNET) versus standardized patient in teaching neurological examinations for the medical students: a randomized, single-blind study. BMC Med Educ. 2021;21(1):493. 34526004 10.1186/s12909-021-02920-4 PMC8444400

[32] Dhar E, Upadhyay U, Huang Y, Uddin M, Manias G, Kyriazis D, et al. A scoping review to assess the effects of virtual reality in medical education and clinical care. Digit Health. 2023;9:20552076231158022. 36865772 10.1177/20552076231158022 PMC9972057

[33] Alharbi A, Nurfianti A, Mullen RF, McClure JD, Miller WH. The effectiveness of simulation-based learning (SBL) on students’ knowledge and skills in nursing programs: a systematic review. BMC Med Educ. 2024;24(1):1099. 39375684 10.1186/s12909-024-06080-z PMC11459713

