# Supplementary material for: Effects of educational intervention on knowledge of Full Outline of Un-Responsiveness score among health workers in uganda: a quasi -experimental pilot study
Source: BMC Med Educ. 2025 Oct 2;25:1298. doi: 10.1186/s12909-025-07839-8 (PMC12492847; doi:10.1186/s12909-025-07839-8)
Supplement: Supplementary file 2 — Supplementary Material 2. [file 12909_2025_7839_MOESM2_ESM.pdf]

Appendix II: Questionnaire

Title of the study: Effects of an educational intervention on knowledge of Full Outline of Un-Responsiveness Score among Health Workers at Mbarara Regional Referral Hospital: A Pilot Study.

Principal Investigator: Obongo Tom

Code……………………………………

Part A: Demographics characteristics of Participant

Instruction: For Question 1 to 9, tick or circle the appropriate response

1. Gender?

a) Male [.]
b) Female [.]

2. What is your Age (Complete years): ……………………………………

3. what is your Profession?

a) Nursing [.]
b) Anaesthesiology [.]
c) Physiotherapy [.]
d) Doctor (MBCHB) [.]
e) Other (specify)……………………………………………………………………………

4. What is your highest level of qualification/education?

a) Certificate [.]
b) Diploma [.]
c) Bachelors/Degree [.]
d) Post-graduate [.], (specify Specialty). ………………………………………………..……....

5. How many years of clinical experience do you have (years); ………………………………….

6. On which ward/unit do you currently work/rotate?

a) Intensive Care Unit (ICU) [.]
b) Accident and Emergency Unit (A&E) [.]
c) Medical-Surgical general ward [.]

7. How long have you rotated in the current unit?

a) Below 3 months

b) 4 to 11 months[.];
c) 1 to 2 years [.];
d) Above 2 years [.].

8. Have you had any training on Full outline of Unresponsiveness (FOUR) score.

a) Yes [.];
b) No [.]

9. If yes to Qtn.8 above, from where?

a) From medical/nursing school [.]
b) In-service training [.]

Part B: Knowledge about Full Outline of Un-Responsiveness (FOUR) score (Question 10-25). Provide your appropriate responses to the following questions below;

10. Define FOUR scale?

…………………………………………………………………………………………………
……………………………………………………………………………………………..

11. What are the four components or parameters of FOUR scale that are monitored during the neurological assessment? Fill in the blank space below (4Mrks)

i. ………………………………………………………………………………………
ii. ………………………………………………………………………………………
iii. ………………………………………………………………………………………
iv. ………………………………………………………………………………………

12. List the components of the Brainstem reflex of the FOUR score? (3 marks)

a) ……………………………………..........................……......
b) ………………………………………………………………...
c)…………………………………………………………………

13. Which of the following statements concerning the indications of the FOUR score is CORRECT?

a) It gives information about level of consciousness only and it is less informative than GCS.
b) It is the most critical assessment parameter to account for possible aphasia, provides information on; brain herniation, respiratory drive, lock-in syndrome, and imminent brain death
c) It does not provide information on prognosis and neurological outcome of patients

14. The total score of the FOUR score ranges from a minimum of 3 in unresponsive patient to the maximum total score of 15 in a patient with full level of consciousness?

a) Yes [.]
b) No [.]

15. The maximum score for motor response in FOUR score is 4, a warded only if the patient demonstrates at least one of the three hand positions; thumb-up, Fist, or peace sign?

a) Yes [.]
b) No [.]

16. Which of the following is the best and safe method recommended for assessing Corneal reflex;

a) Touching the cornea with sterile gloved finger and observing for the reflex [.]
b) Flushing the cornea with torch light and observing for the reflex [.]
c) By instilling 2 to 3 drops of sterile saline held 4 to 6 inches from the cornea [.]
d) By touching the cornea with a piece of gauze and observing for the reflex [.]

17. One of the following is true about the correct sequence of assessing brain stem reflexes;

a) Pupil reflex → cough reflex using tracheal suctioning →corneal reflex
b) corneal reflex → cough reflex using tracheal suctioning → Pupil reflex
c) Pupil reflex → corneal reflex → cough reflex using tracheal suctioning
d) cough reflex using tracheal suctioning → Pupil reflex →corneal reflex

18. Unilateral wide and fixed pupil (Anisocoria) results from pressure exerted on the oculomotor nerve via the tentorial notch.

a) True [.]
b) False [.].

19 ………………………………………………… is a condition where the horizontal eye tracking is absent but the patient has vertical eye tracking or two blinks on command? ***(Fill in the blank 1mrk)***

20. Fill in the blank. When using the FOUR scale, I would award a score of ……… for non-intubated patient with Cheyne-stroke breathing, and ……. for intubated patient triggering the ventilator/breathing above the set ventilator rate (2mrks).

21. Grading of respiratory pattern should be done when the PaCO2 is within the range of?

a) 30-34mmHg,
b) 35-40mmHg,

c) 41-45mmHg,

d) none of the above.

22. In assessing eye response using the FOUR score, the patient's eye must be able to track the finger or an object on command, to obtain a maximum score of 4?

a) Yes [.]

b) No [.]

23. If the eyelids are closed, the examiner should open them and examine tracking of a finger or object?

a) Yes [.]

b) No [.]

24. In case of edema of one eyelid, or facial trauma, tracking with opening of one eye will be sufficient?

a) Yes [.]

b) No [.]

25. Flexion movement of the upper limbs to pain is graded as …. while myoclonus status epilepticus is graded as ……. On FOUR score (2mrks). (**Tick the correct pair**; **M2, M1** [.]; M**3, M0** [.]; **M1, M0** [.], M**2, M0** [.])

THE END

THANK YOU FOR YOU TIME
